# Evaluation of postgraduate family medicine trainee’s knowledge and attitude following an online Educational Module in Palliative Care: A descriptive study

**DOI:** 10.12669/pjms.39.4.6984

**Published:** 2023

**Authors:** Ismat Jabeen, Tayyabah Usman, Munazza Asad, Asra Qureshi, Muhammad Atif Waqar

**Affiliations:** 1 Ismat Jabeen, MBBS, FCPS, MRCGP(Int). Assistant Professor, Department of Family Medicine, Aga Khan University, Karachi, Pakistan; 2 Tayyabah Usman, MBBS Postgraduate Family Medicine Trainee, Year IV Aga Khan University, Karachi, Pakistan; 3 Munazza Asad Postgraduate Family Medicine Trainee, Year IV Aga Khan University, Karachi, Pakistan; 4 Asra Qureshi, Pharm-D, MSc Epidemiology & Biostatistics. Research Instructor, Aga Khan University, Karachi, Pakistan; 5 Muhammad Atif Waqar, MBBS, MD. Assistant Professor, Department of Oncology Aga Khan University, Karachi, Pakistan

**Keywords:** Palliative care, Family Medicine, Education/training

## Abstract

**Objectives:**

To identify improvement in knowledge and attitude of Family Medicine (FM) postgraduate trainees (PGT) towards Palliative care (PC) in order to provide effective care to the patients with advanced disease.

**Methods:**

A cross-sectional study was conducted over eight weeks from 1^st^ July till 3^rd^ September 2021 at Family Medicine Department, Aga Khan University Hospital (AKUH). PGT who willingly signed the written informed consent were enrolled in the study. Descriptive analysis, frequencies, proportions and thematic approach were used for data analysis. Data was analyzed using SPSS version 23.

**Results:**

FM-PGT were included in the study. Improvement in knowledge was observed in posttest scores along with positive change in their attitude and improved perception of level of confidence for managing PC patients. Overall assessment of PCM was positive.

**Conclusion:**

This PCM seems to be a useful tool for PC training in postgraduate medical education (PGME). This highlights some useful aspects for future applications in PC education and training.

## INTRODUCTION

Globally, PC is one of the developing medical subspecialties. World Health Organization (WHO) defines it as an approach that improves the quality of life of patients and their families facing life-threatening illness through prevention, relief of suffering, impeccable assessment and treatment of pain and other symptoms as physical, psycho-social and spiritual.^1^ The Astana Declaration on Primary Health Care (2018) states that “PC must be accessible and available to all.”^2^

In Pakistan, the concept of PC is in stages of infancy, which needs a lot of attention.^3^,^4^ As the burden of disease grows, the need for PC will be enormous.^5^ According to the Global burden of diseases (2010), it is estimated that by 2025 there will be 3.9 million deaths in people aged between 30 to 69 years in Pakistan due to non-communicable diseases including cardiovascular diseases, cancer, diabetes mellitus and mental health illnesses.^6^,^7^ Suffering from such illnesses requires PC.

Family Physicians who are closest to the community and easily accessible have a key role in providing PC.^8^,^9^ It has been revealed that Family Physicians and community nurse teams have taken care of 90% of patients at home in the last 12 months of life. Most of them reported difficulties in pain and other symptoms management, dealing with families’ emotional distress and psychosocial needs with their terminally ill patients.^9^,^10^ It has been found that most young doctors do not have the required knowledge to provide effective end-of-life care.^11^

There are structured PC training programs in developed world, but no such programs exist in Pakistan. Training in PC is challenging, as it has not been incorporated formally into undergraduate and PGT program. It is important to acquire knowledge, skills, and cultural sensitivity to provide long-term and effective PC.^12^ Training needs of FM physicians in PC is vital and cannot be denied.^13^ PC is much underdeveloped in low-middle-income countries and Asia Pacific region and its training is a ray of hope.^14^

FM being the gateway of healthcare and the trainees being uncertain in managing PC patients highlights the need of this study in our population.^15^ Online educational model has gained worldwide fame during Covid-19 pandemic hence affirms the online platform used for this module.^16^-^19^ This module was organized to introduce basic training of PC among FM-PGT. PC being the emerging need of our community and FM-PGT being less confident in managing PC patients as found during their training assessments, mandates the need of this module integration in FM-PGME curricula.

The objective of this study was to highlight this need to improve PC in our population. The results from this study will help develop future PCM modules and training programs. This will provide a model that can be implemented at institutional level or nationally later which would help to address the PC needs. Physicians will be trained in providing basic PC and to reduce unnecessary suffering and improve the quality of life of patients and their families, who are dealing with a life limiting illness. To the best of our knowledge, no such study has been conducted in Pakistan earlier.

## METHODS

This is a descriptive study to evaluate the effectiveness of PCM conducted over eight weeks commencing from 1^st^ July till 3^rd^ September 2021 at FM department of AKUH. FM-PGT who had given written informed consent were enrolled in the study.

### Intervention:

### Phase-I: PCM Development

This module was planned to establish basic concepts of PC based on curricula of Palliative Medicine worldwide. After reviewing literature and discussing with PC specialists, PCM was formulated.

### Phase-II: Implementation

The module was comprised of eight sessions, conducted once per week. This module was facilitated by multidisciplinary PC team including physicians, nurses and dieticians. It consisted of pretest, pre-module need assessment, pre-reading material, MCQs, lectures and case-based discussions. Moodle e-learning platform was used. The module entailed 24 hours of classroom online teaching (synchronous), 24 hours of asynchronous engagement of the participants and two hours for pre-test, posttest, pre-module need assessment and post-workshop evaluation (Total of 50 hours).

### Phase III: Evaluation

Evaluation was done by posttest MCQs, post-module feedback and focus group discussion (FGD). Qualitative exploratory focus group methodology was adopted to gain a wide range of views, stimulate reflection and encourage discussion about PCM. It captured the impact of PCM on the knowledge, attitude, confidence level, experience, strengths and suggestions for improvement.

### Data collection and measures:

All participants were informed about the module plan prior to the commencement of the study. Pre-module need assessment forms and pre-session MCQs were given to all trainees at the beginning of the module and before each session respectively. At the end of the module, PGT were requested to complete post-module feedback form and posttest consisted of MCQs.

The Principal Investigator conducted a FGD at the end of the module, which lasted between 60-90 minutes. A semi-structured interview guide was developed for FGD, supported by the literature and overarching research aims. PGT were asked to share their overall experience and feedback, which was recorded via digital recorder and later converted from voice to text and transcribed. The scores of this study were not included in academic performance assessment. Remedial sessions were arranged for those who scored less than 50%.

### Statistical analysis:

Quantitative data was imported to SPSS version 23 for analysis. Descriptive analysis was done for all non-normally distributed data. Frequencies and proportions of all categorical data was calculated. Descriptive evaluation for performance of PGT in the module and their feedback (pre and post) were performed. The subject expert performed thematic content analysis for the FGD.

### Ethical approval:

Ethical Review Committee of AKU (ERC-AKU) approved the study ethical conduct (ERC No: 2021-6245-18268).

## RESULTS

The total number of PGT participated in PCM were 16. Out of 16, three PGT dropped out due to their rural rotation.

The characteristics of module participants are shown in Table-II. Out of total 13 PGT, n=10(76.92%) was female and n=3(23.07%) was male and year of graduation varied from 2005 to 2019. According to the year of residency, n=5 PGT were from year I, n=5 from year II, n=1 from year III and n=2 from year IV have been enrolled in the study.

**Table-I:** Shows the PCM plan designed for postgraduate Family Medicine trainees.

**Session-1:** Introduction to PC and pain management
**Session-2:** Non-pain symptoms’ management
**Session-3:** Communication and advance care planning
**Session-4:** Role of Nursing
**Session-5:** Ethical and Legal Dimensions
**Session-6:** Nutrition care
**Session-7:** Mental health
**Session-8**: Oncology and PC/ Review Session

**Table:** Table-II:

Characteristics	n (%)
Total no. of residents	13
***Residency level***	***Numbers in each year***
Year I	5
Year II	5
Year III	1
Year IV	2
Year of graduation (MBBS)	2005-2019
***Gender***	
Female	10 (76.9%)
Male	3 (23.07%)

### Pre-module need assessment:

Regarding the objectives, most of the PGT stated that they wanted to learn basics of PC, improve communication skills, find areas of research, provide effective and continuity of care to patients with advanced illness, and refer to appropriate specialty when required. PGT mentioned that learning PC is important because it would help them to manage patients with PC needs. Important themes identified were “symptoms management” “counselling skills”, and “polypharmacy” in PC.

Most of the PGT n=12(92.3%) perceived that PC is about “neither hastening death nor prolonging suffering, while only one n=1(7.69%) of them perceived that it’s about “giving up on active treatment”. Majority of the PGT n=9(69.23%) strongly agreed that PC is important for Family Physicians. While n=4(30.7%) agreed with the statement.

Similarly, n=8(61.53%) PGT strongly agreed that this module would increase their level of confidence in managing patients with advanced illness. While n=5(38.46%) agreed with the given statement.

### Post-module evaluation:

Majority of the participants n= 9(69.23%) agreed that the program objectives were achieved while n=1(7.69%) participants did not agree at all. On other hand, n=3(23.07%) participants agreed that only some objectives have been achieved. The most useful sessions identified by PGT were “symptoms management” and “communication skills”.

The knowledge gained with this module was reported to be excellent by n=4(30.7%), and good by n=8(61.5%) while only n=1(7.69%) participant found it very good.

Level of confidence in managing patients with terminal illness reported by module participants was mostly “good” and “very good”.

PCM organization was reported to be excellent by n=4(30.7%) PGT, very good by n=8(61.5%), while =1(7.69%) reported it as good. Regarding the usefulness of this module in their clinical practice, n=7(53.8%) PGT reported that module was very good, n=5(38.46%) reported good while only n=1(7.69%) reported it to be excellent.

Most of the PGT suggested that module would have been more effective if it could be tailored according to Family Medicine context with more physical and skills sessions including role-plays. The strengths of the module were “communications skills”, “symptoms management”, “learning from the skilled faculty” and “organization of the module” as reported by the PGT. Overall assessment was marked as very good by 50%, excellent by 35.7% and good by 14.29% of study participants.

### Pretest and posttest score:

**Fig.1:**
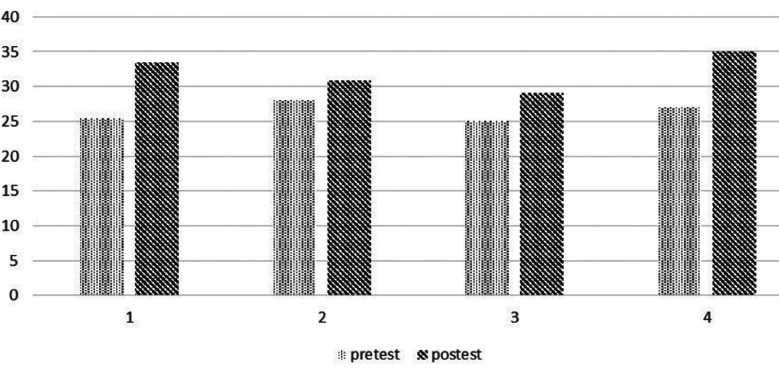
where x-axis shows residency year and y-axis shows pretest and posttest scores.

The pretest score was 61.5% and posttest score was found to be 73.46% with improvement of 12%. The pretest and posttest scores among different residency years are shown in Fig-1. There was increase of knowledge of 12% overall and each level of residency year as shown in the bar chart.

### Focus Group Discussion:

Participants rated PCM as a good experience overall. They appreciated the innovative idea of introducing PCM into Family Medicine core curriculum. The activity was *“well organized”* and it was like *“putting the pieces together”*. Sessions were *“very informative”* and *“helpful”*. Sessions on symptom management and counselling skills were stated as strengths of this module. Some sessions like oncological ailments and nutrition were regarded as beyond the scope of FM. Initially, the perception about PC was end-of-life care but after attending this module, participants understood that it’s about a *“holistic care”* of advanced illnesses with empathy and patients’ well-being physically, socially and mentally. The beauty of PC lies in taking *“care of caregivers”*. Most trainees recognized the need for skills session including breaking bad news and few in-person interactive sessions. Some of them liked the idea of online sessions, as one of the speaker was able to deliver the session from outside the country and those trainees who were on their rural rotations got benefit from that too. Role-plays during the sessions got huge appreciations.

## DISCUSSION

This study emphasizes the vitality of introduction of PCM in curriculum of concerned specialties like FM which will enable physicians to take care of this huge vulnerable population of our country. The results of this study reveals improvement of knowledge by 12%, increased level of confidence in managing patients with advanced diseases and overall assessment for the module was marked as good and very good by most of the participants. These outcomes are comparable to other studies for online educational intervention, such as those reported about symptoms management at the end-of-life.^17^-^25^ Most of the participants assigned a score of “good and very good” to the usefulness of educational content in their clinical practice. The content and organization of the module was well-received.

Education using an e-learning platform had a positive impact as mentioned by a few participants. Although majority of the participants expressed the need of more physical and skills sessions including role-plays, but also realized the need of the online platform to facilitate the trainees on their rural and elective rotations. Further studies and skill-oriented training programs with long-term follow up evaluations may address these issues.

The findings of this study reflect the educational and training need of the PGT regarding PC. Family Physicians being the first point of care for several people can provide effective care if trained properly, so training need is of utmost importance. This module can be a useful resource for PC physicians and experts to develop capacity of family physicians in the field of Palliative medicine and should be offered to other physicians interested in PC education soon. There is a need to design further educational programs and interventions and evaluate their impact on patients, families and health care providers on local, regional and national levels.

### Limitations:

The study sample size was small. Due to Covid-19 pandemic and unavailability of few PGT, all the sessions were conducted online. Skills sessions could not be conducted physically as well.

## CONCLUSION

This online module on PC had positively influenced PGT in this study by bringing a significant change in their knowledge and attitude. This module can be a useful resource for PC Physicians and experts to develop capacity of Family Physicians in the field of Palliative Medicine and should be offered to other physicians interested in PC education in the near future. There is a need to design further educational programs and interventions and evaluate their impact on patients, families and health care providers on local, regional and national levels.

### Abbreviations:

**Table:** 

**PC**	Palliative Care
**FM**	Family Medicine
**PGT**	Postgraduate trainees
**AKUH**	Aga Khan University Hospital
**PCM**	Palliative Care Module
**FGD**	Focus group discussion

### Authors’ Contributions:

**IJ:** Principal Investigator and Corresponding Author; Proposed original idea, developed study design and questionnaire, contributed to manuscript writing and responsible for accuracy of work. Conducted focus group interview.

**TU:** Contributed to data collection, scholarly writing including background and discussion. Helped in organization of online educational platform.

**MA:** Contributed to scholarly writing including methodology, tabulation of results and data entry. Helped in transcribing focus group discussion.

**AQ:** Performed statistical analysis.

**MAW:** Senior Author: helped in proofread final version of manuscript.
